# Seroprevalence of anti-*Toxocara canis* antibodies and associated risk factors among dog owners in the rural community of Nakhon Si Thammarat province, southern Thailand

**DOI:** 10.1186/s41182-022-00425-4

**Published:** 2022-05-17

**Authors:** Prasit Na-Ek, Udomsak Narkkul, Nonthapan Phasuk, Chuchard Punsawad

**Affiliations:** 1 Department of Medical Science, School of Medicine, Walailak University, Nakhon Si Thammarat, 80160 Thailand; 2 Research Center in Tropical Pathobiology, Walailak University, Nakhon Si Thammarat, 80160 Thailand; 3 Department of Medical Clinical Science, School of Medicine, Walailak University, Nakhon Si Thammarat, 80160 Thailand

**Keywords:** Seroprevalence, *Toxocara canis*, Dog owner, Thailand

## Abstract

**Background:**

Human toxocariasis is a zoonotic parasitic disease caused by the *Toxocara canis* and *T. cati* nematodes larvae. Dog owners are at a higher risk of acquiring *T. canis* infection, and there is no available evidence regarding the seroprevalence of *T. canis* infection among dog owners in Thailand. Therefore, this study aimed to investigate the seroprevalence of *T. canis* infection and associated risk factors among dog owners in rural areas of Thailand.

**Methods:**

A total of 132 dog owners, including 25 men and 107 women, were recruited for this study. Serum anti-*T. canis* IgG antibodies were detected using a commercial enzyme-linked immunosorbent assay (ELISA) kit, and information on risk factors was collected using a questionnaire. In addition, hematological parameters were analyzed by the auto hematology analyzer. Risk variables associated with *T. canis* infection were investigated using univariate and multivariate logistic regression models.

**Results:**

The overall seroprevalence of *T. canis* was 76.5% (101/132). Men were more likely to be infected with *T. canis* than women. Univariate analysis revealed that dog owners who did not practice handwashing before meals (*p* = 0.005) or after contact with soil (*p* = 0.035) or dogs (*p* = 0.049) had a substantially higher risk of acquiring *T. canis* infection. After adjusting for confounders, not practicing handwashing before meals remained a significant risk factor for *T. canis* infection (*p* = 0.038). The mean number of eosinophils was significantly higher in the seropositive group than in the seronegative group.

**Conclusions:**

This is the first serological report of *T. canis* infection among dog owners reflecting the high rate of *T. canis* seropositivity in rural areas of southern Thailand. This study also provides group-specific data concerning modifiable risk behaviors for more effective *T. canis* infection control and prevention strategies in Thailand.

**Supplementary Information:**

The online version contains supplementary material available at 10.1186/s41182-022-00425-4.

## Background

Toxocariasis in humans is a zoonotic disease caused by the parasitic roundworm of the genus *Toxocara,* particularly prevalent in tropical and subtropical areas and developing countries [1, 2]. The domestic dog is the definitive host for *Toxocara canis* and the domestic cat for *T. cati* [3]. Reportedly, the global seroprevalence of *T. canis* infection is 11.1% in dogs [4]. In humans, toxocariasis is caused by accidental ingestion of *Toxocara* embryonated eggs containing larvae, which are contaminated in the soil by dog or cat feces, or via the consumption of larvae worms in raw or poorly cooked meat by paratenic hosts infected with *Toxocara* larvae [5]. In humans, the estimated global seroprevalence of toxocariasis is 19.0% [6].

Although most patients infected with *Toxocara* are asymptomatic, four clinically symptomatic forms of human toxocariasis have been reported: Common toxocariasis (covert toxocariasis), visceral larva migrans (VLM), ocular larva migrans (OLM), and neurotoxocariasis [2]. However, *Toxocara* infection also contributes to the development of allergic diseases such as asthma, which is globally prevalent [7]. *Toxocara* is unable to complete its lifecycle in humans; hence, no *Toxocara* eggs have been found in the human stool [2, 8]. The gold standard test for diagnosing toxocariasis is the identification of the organism using microscopy, which is seldom performed for practical reasons. Therefore, an enzyme-linked immunosorbent assay (ELISA) that detects IgG-specific antibodies against *T. canis* excretory-secretory (TES) antigens is widely available and recommended by the Centers for Disease Control and Prevention [9]. However, TES-based immuno-diagnosis is limited because of the cross-reactivity of *Toxocara* TES antigens with other roundworms, especially human *Ascaris lumbricoides,* and this test is also incapable of differentiating between active and previous *Toxocara* infections.

The seroprevalence of *T. canis* has been reported in several countries. Regions with high seroprevalence of toxocariasis include the rural areas of Rio, Brazil (71.8%) [10], Makoko, Nigeria (86.1%) [11], and the Republic of the Marshall Islands (86.7%) [12]. In Western countries, the seroprevalence of toxocariasis is low, including the United States (5.1%) [13], Mexico (4.7% in adults and 13.8% in children) [14], Italy (8%) [15], and Greece (16%) [16]. In Southeast Asia, there are many countries that have a slightly higher seroprevalence, such as Nakhon Si Thammarat Province, Thailand (58.2%) [17], Ho Chi Minh City, Vietnam (45.2%) [18], and Los Baños, Laguna, Philippines (49%) [19]. Dogs are popular pets in many countries around the world and are beneficial for owners in several ways, such as security, improved mental health, socialization, and physical wellbeing [20]. Infection with *Toxocara* spp. in dogs has been reported worldwide, including in the Eastern Mediterranean region, Africa, and Southeast Asia (including Thailand), North America, South America, Europe, and the Western Pacific [21–24]. Dog owners are prone to be infected with *T. canis* from their dogs by ingesting animal fecal material. Moreover, close contact, lack of handwashing, and increased risk of pet-associated disease have been reported [6, 17]. However, few studies have explored the seroprevalence of *T. canis* in dog owners.

Currently, there are no reports on the seroprevalence of *T. canis* infection in dog owners living in the rural areas of southern Thailand. Therefore, this study aimed to investigate the seroprevalence of *T. canis* infection and its associated risk factors among dog owners in the rural areas of Nakhon Si Thammarat Province, Thailand.

## Methods

### Study site and setting

**Fig. 1:**
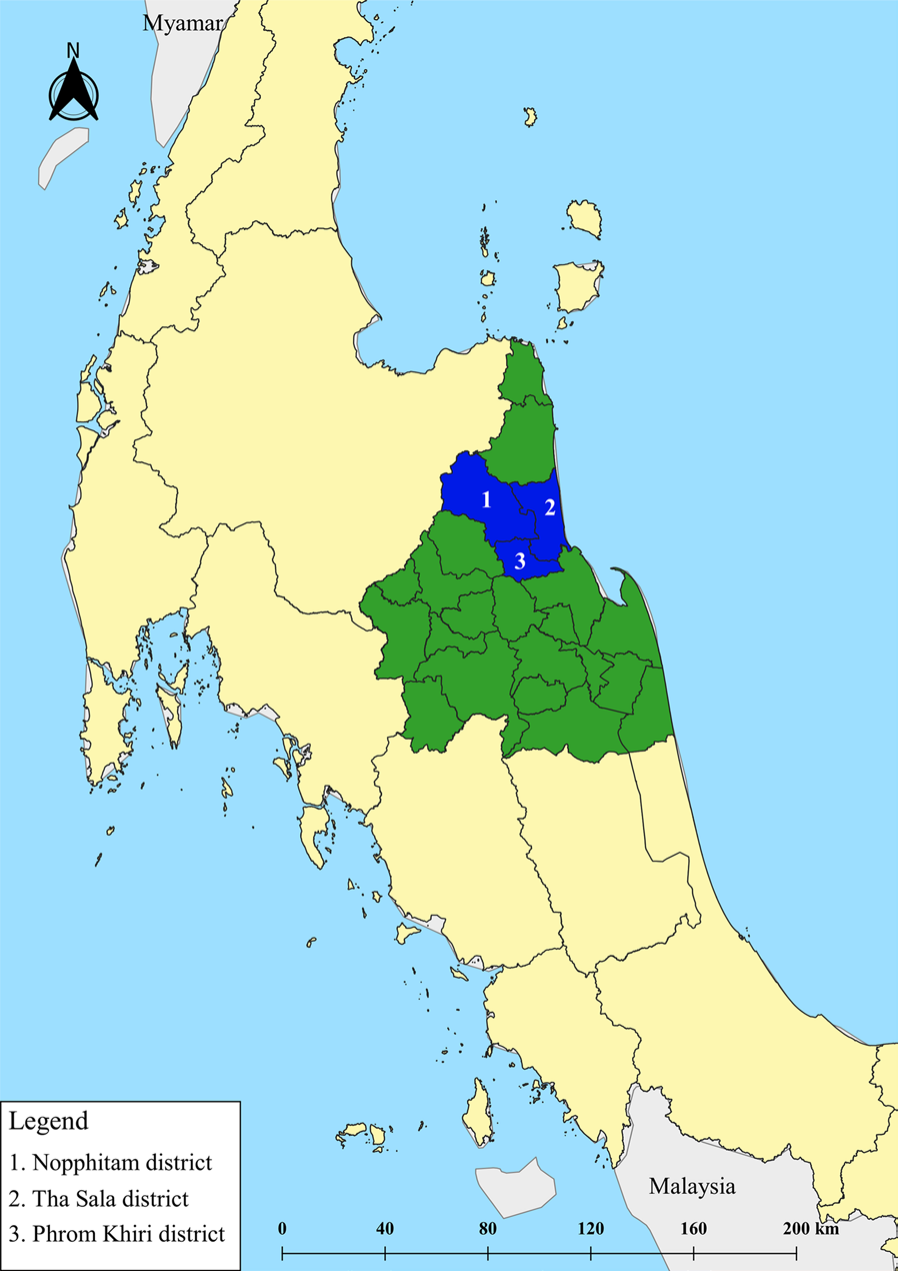
Map of Nopphitam (1), Tha Sala (2), and Phrom Khiri (3) Districts as study sites. The map was generated using Quantum GIS version 3.16.11 (ESRI basemaps)

The study was carried out from September to November 2020 in three districts of Nakhon Si Thammarat Province: Tha Sala, Phrom Khiri, and Nopphitam (Fig. 1). The Nakhon Si Thammarat Province is located in southern Thailand, approximately 780 km from Bangkok (8° 25′ 7″ N, 99° 57′ 49″ E). The average temperatures are 27.7 °C, 27.1 °C, and 26.6 °C in September, October, and November, respectively. The average rainfall from September to November 2020 was 429.4 mm, and the cumulative rainfall was 1288.2 mm (Climatological Center, Thai Meteorological Department). The Thailand Department of Provincial Administration estimated in 2019 that the total population in Tha Sala was 118,113, Phrom Khiri was 37,469, and Nopphitam was 33,533. All districts were similar in terms of culture, economic status, and climate.

### Population of the study and sample size

The study population consisted of dog owners > 18 years from different houses in the three districts of Nakhon Si Thammarat Province. The sample size was determined using a single-proportion population as follows:

1 $$Z^{2} {{p\left( {1 - p} \right)} \mathord{\left/ {\vphantom {{p\left( {1 - p} \right)} {d^{2} }}} \right. \kern-\nulldelimiterspace} {d^{2} }}$$

where *p* is the prevalence of intestinal parasites from a previous study, *d* is the margin of error, and *Z* is the standard score, which corresponds to 1.96. This formula was calculated based on a prevalence rate of 9.3% from a previous study [23], with a margin of error of 0.05 and a confidence level of 95%. The calculated sample size was 130. The exclusion criteria were immune system disorders (such as autoimmune disorders and acquired immune deficiency syndrome), steroid treatment for at least 3 months, and acute illnesses on the day of blood collection. In total, 132 dog owners were randomly selected from each district using a systematic random sampling technique in which every third person was selected for the study.

### Questionnaire and survey

The questionnaire consisted of 19 close-ended questions and one open-ended question (Additional file 1) to collect information on the general demographic data of the dog owner (gender, age, district, religion, occupation, and education), information on the dog (number of dogs, species, health check/vaccination, deworming, shower, and clearing ticks and fleas), and information on possible risk factors (playing with the dog; kissing or touching the dog; location of feces discharge; sleeping spot; feces management; and habits of handwashing before a meal, after soil contact, and after contact with the dog). The occupations were divided into 2 groups, including unskilled and skilled workers. Unskilled workers refer to workers with no particular skills or no formal education, including agriculturists, farmers, merchants, and housemaids. Skilled workers mean workers with specialized training or learned skill sets, including officials and teachers. Two professional interviewers conducted direct interviews with the participating dog owners to administer the questionnaire.

### Blood collection and preparation

Medical technologists collected blood samples (5 mL) from the antecubital vein. The collected blood samples (3 mL) were immediately separated into clot blood tubes, which were used to measure IgG class antibodies against *T. canis*. The blood samples were kept at 4 °C in a cooling box and were immediately transferred to the laboratory for serum preparation. Serum samples were kept at − 80 °C until further examination. Another 2 mL of collected blood was added to an EDTA tube, stored at 4 °C, and transported immediately to the laboratory to measure hematological parameters.

### Analysis of hematological parameters

Hematological parameters, including red blood cells (RBC), hemoglobin (Hb), hematocrit (HCT), mean cell volume (MCV), mean corpuscular hemoglobin (MCH), mean corpuscular hemoglobin concentration (MCHC), red cell distribution width (RDW), white blood cells (WBC), neutrophils (Neu), lymphocytes (Lymp), eosinophils (Eo), monocytes (Mono), basophils (Baso), and platelets (PLT), were analyzed by the auto hematology analyzer at the Medical Technology Clinic, Walialak University.

### Detection of anti-*Toxocara canis* IgG antibodies

Commercial ELISA kits (NovaLisa® (NOVTOCG0450), NovaTec Immunodiagnostica GmbH, Dietzenbach, Germany) with > 95% sensitivity and specificity were used to measure serum anti-*T. canis* IgG antibodies, according to the manufacturer’s protocol. In brief, all samples were diluted 1 + 100 with IgG sample diluent, and all controls (*T. canis* IgG-positive, *T. canis* IgG-negative, *T. canis* IgG cutoff, and substrate blank) were prepared. The following requirements must be satisfied for an assay to be considered valid: cutoff was 0.150–1.300, negative controls were < 0.200 and < cutoff, positive controls were > cutoff, and the substrate blank was < 0.100. Next, 100 μL of the control or diluted sample was added to pre-coated 96-well plates and incubated for 1 h at 37 °C. After incubation, the plates were washed three times with washing buffer, and all wells, except for the substrate blank well, were incubated with 100 μL of protein A horseradish peroxidase (HRP) conjugate for 30 min at room temperature. The plates were washed three times and incubated with 100 μL 3,3′,5,5′-Tetramethylbenzidine (TMB) substrate solution for 15 min at room temperature in the dark. Next, the stop solution (100 μL) was added to each well and incubated for 15 min at room temperature to stop the reaction. The assay included negative and positive serum samples (provided by the ELISA kits) and a substrate blank in addition to a blank (no serum sample). The absorbance was measured at 450/620 nm using an automatic microplate reader. For interpretation, the results were calculated to NovaTec units (NTU), samples with > 11 NTU were considered positive. However, if the NTU value was between 9 and 11, the sample was considered equivocal and a fresh sample was repeated. If the results of the repeated test were also equivocal, the sample was considered negative.

### Statistical analysis

All data were entered, cleaned, and analyzed using the SPSS software (version 17.0; SPSS Inc., Chicago, IL, USA). The mean and standard deviation (SD) were used to describe quantitative data, whereas frequencies (percentages) were used to describe qualitative data. A Chi-squared test was used to compare the proportions of *T. canis* infections among subgroups stratified by sex, age, district, religion, occupation, and education. Risk variables associated with *T. canis* infection were investigated using a univariate logistic regression model. The variables in the univariate logistic regression model with *p* < 0.1 were included in a multiple logistic regression model that was adjusted for confounding factors. Differences were considered statistically significant when *p* < 0.05. The hematological parameters of seropositive and seronegative dog owners were compared using the Mann–Whitney *U* test and independent *t* test for nonparametric and parametric data analysis, respectively.

## Results

### Characterization of sociodemographic factors

This study included 132 dog owners, including 25 men (18.94%) and 107 women (81.6%). The median age of the participants was 50 years (interquartile range 20 years). The participants lived in three districts of Nakhon Si Thammarat Province: Tha Sala (*n* = 53), Nopphitam (*n* = 37), and Phrom Kiri (*n* = 42). All participants were Buddhists. Their occupations and educational levels predominantly were unskilled workers and high school diplomas or lower, respectively. The mean number of dogs per household was 1.9. All the participants allowed their dogs to excrete outside the house (Table 1).

**Table 1:** Sociodemographic characteristics of 132 dog owners in this study

Group	Number	%
Total	132	
Gender		
Male	25	18.9
Female	107	81.1
Age group (years)		
18–35	20	15.1
36–53	63	47.7
54–71	36	27.3
72–89	13	9.9
District		
Thasala	53	40.2
Nopphitam	37	28.0
Phrom Kiri	42	31.8
Religion		
Buddhists	132	100.0
Occupation		
Unskilled workers	129	97.7
Skilled workers	3	2.3
Education		
High school or less	122	92.4
Bachelor’s degree or more	10	7.6
Dog owner’s behavior		
Dog excretion outside the house	132	100.0

### Seroprevalence of *Toxocara canis* infection

The seroprevalence of *T. canis* infection among dog owners is shown in Table 2. The results showed that the overall seroprevalence of *T. canis* infection was 76.5%. Male participants (80%) had a higher seropositivity rate than female participants (75.7%). The rates of seropositivity were 69.4%, 89.2%, and 73.8% in the Tha Sala, Nopphitam, and Phrom Kiri districts, respectively, but were not significantly different among the three districts (*p* = 0.910). Seropositivity for *T. canis* was observed in all age groups. The highest rate of seropositivity was found among 18–35 years (85%), then 72–89 years (76.9%), whereas the lowest rate of seropositivity was observed among 36–53 years (74.6%). Chi-squared tests showed no significant associations between *T. canis* seropositivity and sex, age, and education (Table 2).

**Table 2:** Seroprevalence of *Toxocara canis* infection among 132 dog owners in this study

Group	Number	Seropositivity	%	*p* value
Total	132	101	76.5	
Gender				
Male	25	20	80.0	0.796
Female	107	81	75.7	
Age group (years)				
18–35	20	17	85.0	
36–53	63	47	74.6	
54–71	36	27	75.0	
72–89	13	10	76.9	0.807
District				
Thasala	53	37	69.8	0.910
Nopphitam	37	33	89.2	
Phrom Kiri	42	31	73.8	
Occupation				
Unskilled workers	129	99	76.7	1.000
Skilled workers	3	2	66.7	
Education				
High school or less	122	94	77.0	0.699
Bachelor’s degree or more	10	7	70.0	

### Risk factors analysis

Table 3 shows the summary of findings from the regression analysis to investigate the associations between *T. canis* infection and potential factors. The univariate analysis revealed that dog owners who did not wash their hands before meals (crude odds ratio (COR) = 8.383; 95% CI 1.89–37.16; *p* = 0.005), after contact with soil (COR = 5.027; 95% CI 1.12–22.54; *p* = 0.035), and after contact with the dog (COR = 4.519; 95% CI 1.00–22.34; *p* = 0.049) had a significantly increased risk of acquiring *T. canis* infection. However, after adjusting for confounders, only dog owners who did not wash their hands before meals remained at significant risk of acquiring *T. canis* infection (adjusted odds ratio (AOR) = 6.067; 95% CI 1.10–33.34; *p* = 0.038). Dog owners who did not wash their hands before meals were 6.067 times more likely to have acquired *T. canis* infection than those who had not after adjusting for other factors. When stratified by age, the rates of seropositivity for *T. canis* were approximately equal among the age groups (74.6%, 75%, 76.9%, and 85% for dog owners aged 36–53, 54–71, 72–89, and 18–35 years, respectively). Men were more likely to be infected with *T. canis* than women. However, there were no statistically significant differences among these factors in the univariate analysis (Table 3). Univariate analysis of the other information on the dogs and the dog owner’s behavior, including bleeding in the dog; vaccination of the dog; deworming of the dog; bathing the dog; tick/flea treatment; behavior of dog owner regarding playing, kissing, and touching the dog; the sleeping place of the dog; frequency of playing with the dog; and management of the dog feces, showed no statistically significant differences (Table 3).

**Table 3:** Factors associated with *Toxocara canis* infection among dog owners

Variables	Number (%)	Seropositive (%)	Seronegative (%)	COR	95% CI	*p* value	AOR	95% CI	*p* value
Gender									
Male	25 (18.9)	20 (80.0)	5 (20.0)	1					
Female	107 (81.1)	81 (75.7)	26 (24.3)	1.284	0.44–3.76	0.649			
**Age (years)**									
18–35	20 (15.1)	17 (85.0)	3 (15.0)	1					
36–53	63 (47.7)	47 (74.6)	16 (25.4)	0.518	0.13–2.00	0.341			
54–71	36 (27.3)	27 (75.0)	9 (25.0)	0.529	0.12–2.24	0.387			
72–89	13 (9.9)	10 (76.9)	3 (23.1)	0.588	0.10–3.49	0.559			
Dog bleed									
Local bleed	107 (81.1)	82 (62.1)	25 (18.9)	1					
Foreign bleed	13 (9.8)	3 (2.3)	10 (7.6)	0.869	0.21–3.67	0.848			
Hybrid bleed	12 (9.1)	9 (6.8)	3 (2.3)	1.105	0.16–7.49	0.919			
Dog vaccination									
Yes	63 (47.7)	46 (34.8)	17 (12.9)	1					
No	69 (52.3)	55 (41.7)	14 (10.6)	1.03	0.40–2.63	0.951			
Dog deworm									
Yes	41 (31.1)	29 (22.0)	12 (9.1)	1					
No	91 (68.9)	72 (54.5)	19 (14.4)	1.122	0.43–2.95	0.815			
Bathing dogs									
Yes	80 (60.6)	59 (73.8)	21 (26.2)	1					
No	52 (39.4)	42 (80.8)	10 (19.2)	1.161	0.45–2.95	0.757			
Get rid of tick/fleas									
Yes	93 (70.5)	67 (72.0)	26 (28.0)	1					
No	39 (29.5)	34 (87.2)	5 (12.8)	2.442	0.75–8.00	0.140			
Play with dogs									
Yes	95 (72.0)	72 (75.8)	23 (24.2)	1.158	0.46–2.88	0.753			
No	37 (28.0)	29 (78.4)	8 (21.6)	1					
Kiss with dogs									
Yes	30 (22.7)	23 (76.7)	7 (23.3)	0.989	0.38–2.59	0.982			
No	102 (77.3)	78 (76.5)	24 (23.5)	1					
Touch with dogs									
Yes	98 (74.2)	75 (76.5)	23 (23.5)	0.997	0.40–2.50	0.994			
No	34 (25.8)	26 (76.5)	8 (23.5)	1					
Sleep place									
Indoor	12 (9.1)	11 (91.7)	1 (8.3)	1					
Outdoor	120 (90.9)	90 (75.0)	30 (25.0)	0.273	0.03–2.20	0.223			
Frequency of playing with dogs									
Never	36 (27.3)	28 (77.8)	8 (22.2)	1					
1–2 times/week	40 (30.3)	29 (72.5)	11 (27.5)	0.75	0.23–2.44	0.633			
3–4 times/week	22 (16.7)	16 (72.7)	6 (27.3)	0.561	0.18–1.74	0.319			
5–7 times/week	34 (25.8)	28 (82.4)	6 (17.6)	0.571	0.16–2.07	0.394			
Handwashing before meals									
Yes	112 (84.8)	83 (74.1)	29 (25.9)	1			1		
No	20 (15.2)	18 (90.0)	2 (10.0)	8.383	1.89–37.16	0.005^*^	6.067	1.10–33.34	0.038^*^
Handwashing after contact with soil									
Yes	111 (84.1)	82 (73.9)	29 (26.1)	1			1		
No	21 (15.9)	19 (90.5)	2 (9.5)	5.027	1.12–22.54	0.035^*^	2.096	0.38–11.40	0.392
Handwashing after contact with dog									
Yes	108 (81.8)	80 (74.1)	28 (25.9)	1			1		
No	24 (18.2)	21 (87.5)	3 (12.5)	4.519	1.00–22.34	0.049^*^	1.082	0.17–6.78	0.933
Dog feces management									
Yes	66 (50.0)	49 (74.2)	17 (25.8)	1					
No	66 (50.0)	52 (78.8)	14 (21.2)	1.289	0.57–2.89	0.538			

*Statistically significant at *p* < 0.05

### *Toxocara canis* infection and hematological parameters

The hematological parameters for the *T. canis* IgG seropositive and *T. canis* IgG seronegative groups are displayed in Table 4. The results revealed that the mean number of eosinophils was significantly higher in the *T. canis* IgG seropositive group than in the seronegative group (*p* < 0.04). There were no significant differences in RBC count, MCHC, Hb, HCT, MCV, RDW, WBC, neutrophil number, lymphocyte number, monocyte number, or basophil number between the two groups (Table 4).

**Table 4:** Comparison of hematological parameters between *Toxocara canis* IgG seropositive and *Toxocara canis* IgG seronegative groups

Blood parameters	Normal ranges	Groups		*p* value
		Seropositive (*n* = 101)	Seronegative (*n* = 31)	
		Mean ± SD	Mean ± SD	
RBC (10^6/ul)^a^	3.75–6.54	4.68 ± 0.43	4.59 ± 0.6	0.45
Hb (g/dL)^b^	12.00–16.00	12.66 ± 1.76	12.51 ± 2.1	0.53
Hct (%)^b^	36.00–48.00	39.61 ± 4.63	39.13 ± 6.21	0.56
MCV (fL)^a^	80.00–99.00	84.82 ± 9.14	85.23 ± 8.29	0.88
MCH (pg)^a^	27.00–31.00	27.22 ± 3.56	27.32 ± 2.94	0.94
MCHC (d/dL)^a^	33.00–37.00	32.01 ± 1.64	31.94 ± 1.21	0.66
RDW (%)^b^	11.50–14.50	13.26 ± 1.76	13.48 ± 1.59	0.14
WBC (cells/cu.mm.)^a^	4000–10,000	8017.72 ± 2202.69	7380.32 ± 2014.73	0.13
Neu (cells/cu.mm.)^a^	1000–8000	4316.4 ± 1463.59	4002.1 ± 1282.32	0.28
Lymp (cells/cu.mm.)^a^	1500–7000	2818.88 ± 783.93	2643.2 ± 908.5	0.30
EO (cells/cu.mm.)^a^	< 500	516.45 ± 436.09	344.07 ± 197.82	0.04^*^
Mono (cells/cu.mm.)^a^	200–1000	315.45 ± 143.87	346.32 ± 223.08	0.94
Baso (cells/cu.mm.)^a^	< 210	50.54 ± 44.04	44.63 ± 46.67	0.52
PLT (cells/cu.mm.)^b^	140,000–450,000	234,148.51 ± 62,329.99	220,064.52 ± 59,432.84	0.36

^a^Data analysis by independent t-test

^b^Data analysis by Mann–Whitney U

*Significant differences were identified at *p* < 0.05

## Discussion

Toxocariasis in humans is a neglected zoonotic parasite that affects millions of pediatric and adolescent populations globally, especially in tropical and subtropical regions. This is the first serological investigation of *T. canis* infection among dog owners in rural areas of southern Thailand. The present study showed that the overall seroprevalence of *T. canis* infection was 76.52%, which was higher than that reported in Iran (20.43%) [25] and Egypt (29.85%) [26]. In addition, the results of our study were also higher than the globally reported seroprevalence (19.0%) and the pooled seroprevalence in Southeast Asia (34.1%) [6], Thailand (58.2%) [17], Vietnam (45.2%) [18], and the Philippines (49%) [19]. However, the seroprevalence rate in this study was lower than that in other regions, including Makoko, Nigeria (86.1%) [11], and the Republic of the Marshall Islands (86.75%) [12]. Several factors contribute to *T. canis* infection, including geographic, socioeconomic, climatic, environmental, and cultural factors. The present study covered three districts of Nakhon Si Thammarat Province, and all areas were considered rural communities. These countryside areas are filled with palm oil and rubber plantations. Nakhon Si Thammarat Province has a tropical rainforest climate with temperatures ranging from 24 to 34 °C, suitable for the development of the infective larval stage of *Toxocara* eggs. Dogs are the most popular pets in this province, with a large number of stray dogs in the area. The mutual affection of humans and their companions, accepted as an operative connection known as the human–animal bond, provide several benefits with regard to mental health, socialization, and physical wellbeing [27]. However, privately owned dogs can play a crucial role in helminthic zoonotic toxocariasis transmission [28, 29]. We hypothesized that the soil, number of dogs in the areas, and the human–animal bond influenced the seropositivity rate in this study.

Among the participating dog owners in the three districts, the highest *T. canis* seropositivity (89.2%) was observed in the Nopphitam District. However, there were no statistically significant differences between the three districts. This might be explained by the similarities in the areas, such as climate, geography, values, and culture among the districts. Most dog owners who were unskilled workers with low education levels had a relatively higher rate of *T. canis* infection. This correlation was similarly observed in a previous study conducted in a rural area of southern Thailand [17]. However, this study revealed that educational level and occupation did not significantly influence the seropositivity rate of *T. canis*. This might be due to the small number of dog owners who were skilled workers and had obtained college degrees. While comparing age groups, our study showed no significant difference in the seropositivity rate of *T. canis* infection among different age groups. The seropositivity rates of *T. canis* infection were similar across all age groups: 74.6% in dog owners aged 36–53 years, 75% in those aged 54–71 years, 76.9% in those aged 72–89 years, and 85% in those aged 18–35 years. However, this result was in contrast to several studies that showed that older age was a significant risk factor for *T. canis* infection [11, 13, 30, 31]. Serum *Toxocara* IgG can persist with age, which increases the detection of *Toxocara* via serology over time. A possible explanation for these similar seropositivity rates among different age groups may be that the dog owners came in contact with *T. canis* eggs at a young age, as IgG persists over a long period of life. Our study revealed that men (80%) were more likely to be infected with *T. canis* than women (75.7%). This trend has also been reported in previous studies [31, 32]. It has been hypothesized that males engage in more outdoor activities than females and, therefore, are more likely to be in contact with contaminated soil and dogs [33, 34]. However, univariate and multivariate analyses demonstrated that sex was not significantly associated with seropositivity for anti-*T.canis* IgG antibodies in our study.

Regarding modifiable risk behaviors, handwashing before meals appeared to be the only significant risk factor associated with *T. canis* infection in both the univariate and multivariate analyses. Dog owners who did not practice handwashing before meals were six times more likely to be infected with *T. canis* than those who did. The risk ratio in this study was higher than that in a previous study conducted on schoolchildren in Nakhon Si Thammarat Province, Thailand [17]. Interestingly, the univariate analysis in this study revealed that not practicing handwashing after coming in contact with soil and dogs was significantly associated with *T. canis* infection. However, these associations were not statistically significant in the multivariate analysis. A similar result was observed in a previous study [17]. Humans acquire *T. canis* by accidentally ingesting infected eggs, which can be found in several public places [35]. A previous study conducted in rural areas of southern Thailand reported that the soil samples from public schools were contaminated with *Toxocara* eggs, especially that in the playgrounds; this correlated with our results as all dog owners reportedly allowed the dog to excrete outside the house (100%) [34]. This study suggests that people might be infected with *T. canis* in ways other than by caring for dogs and emphasizes the necessity of hand hygiene to prevent parasite eggs from entering the human body via the fecal–oral route. Further investigation for the presence of *Toxocara* eggs in dogs’ feces and soil samples is needed to understand the potential etiological factors of the disease transmission to humans.

The results of the hematological profile showed a higher eosinophil count in the seropositive group than in the seronegative group. This finding is similar to those of previous studies on other parasites [36, 37], suggesting that eosinophils play a major role in the immune response against tissue parasites. These results are similar to the previous study in which eosinophil appeared to increase during hookworm infection [37].

The study had several limitations. First, ELISA is a method for detecting anti-*T. canis* IgG antibodies, which is not the reference laboratory test for *T. canis* detection, *Toxocara* spp*.* larval excretory-secretory antigens Western blot, and could yield false-positive results due to cross-reactivity with other helminths, especially *A. lumbricoides* [38, 39]. However, our study area was not endemic to *A. lumbricoides* [40–42]. Consequently, the probability of false positivity is likely minimal. Second, the study performed cross-sectional research, and the seroprevalence and the risk factors were evaluated concurrently and not over a period of time; hence, the true causes and effects might not be strongly determined.

## Conclusions

This is the first serological investigation of *T. canis* infection among dog owners in southern Thailand with a high rate of *T. canis* seropositivity, reflecting high levels of *T. canis* exposure among dog owners in this region. Not practicing handwashing before meals appeared to be a significant risk factor for *T. canis* infection. This study also provides group-specific data concerning modifiable risk behaviors for more effective *T. canis* infection control and prevention strategies in Thailand.

## Supplementary Information

**Additional file 1.** Questionnaire Form.

## Data Availability

All data generated or analyzed during this study are available from the corresponding author on reasonable request.

## Abbreviations

VLM: Visceral larva migrans
OLM: Ocular larva migrans
ELISA: Enzyme-linked immunosorbent assay
TES: *T. canis* excretory–secretory
RBC: Red blood cells
Hb: Hemoglobin
HCT: Hematocrit
MCV: Mean cell volume
MCH: Mean corpuscular hemoglobin
MCHC: Mean corpuscular hemoglobin concentration
RDW: Red cell distribution width
WBC: White blood cells
NTU: NovaTec units
