# Supplementary material for: Seroprevalence of anti-Toxocara canis antibodies and associated risk factors among dog owners in the rural community of Nakhon Si Thammarat province, southern Thailand
Source: Trop Med Health. 2022 May 17;50:32. doi: 10.1186/s41182-022-00425-4 (PMC9112435; doi:10.1186/s41182-022-00425-4)
Supplement: Supplementary file 1 — Additional file 1. Questionnaire Form. [file 41182_2022_425_MOESM1_ESM.docx]

**Supplementary Materials 1: Questionnaire Form**

**Part 1:** General information

1. Gender 🔾 1. Male 🔾 2. Female
2. Age ............. years
3. District

🔾 1. Tha Sala 🔾 2. Phrom Khiri 🔾 3. Nopphitam

1. Religion 🔾 1. Buddhism 🔾 2. Christian

🔾 3. Islam 🔾 4. Other…………………

1. Occupation

🔾 1. Agriculturists/farming 🔾 2. Company employee

🔾 3. Freelance 🔾 4. Officialdom

🔾 5. Other…………………

1. Education

🔾 1.Primary school 🔾 2. Secondary school

🔾 3. Vocational certificate 🔾 4. Bachelor Degrees

🔾 5. Master Degrees or higher 🔾 6. Other…………………

**Part 2:** Dog information, dog owner possible risk factors and personal hygiene

1. Dog bleed/species

🔾 1. Local bleed 🔾 2. Foreign bleed

🔾 3. Hybrid bleed

1. Did you do health check/vaccination for a dog every 1 month?

🔾 1. Yes 🔾 2. No

1. Did you do health deworm for a dog every 3 month?

🔾 1. Yes 🔾 2. No

1. Did you clean/bath for a dog every week?

🔾 1. Yes 🔾 2. No

1. Did you get rid of tick/flea for a dog every week?

🔾 1. Yes 🔾 2. No

1. How often you play with dog?

🔾 Never 🔾 1–2 times/week

🔾 3–4 times/week 🔾 5–7 times/week

1. Did you kiss with dog?

🔾 1. Yes 🔾 2. No

1. Did you ever touch with dog?

🔾 1. Yes 🔾 2. No

1. Where your dog excretion place?

🔾 Inside houses 🔾 Outside the house

1. Where your dog sleep?

🔾 Inside houses 🔾 Outside the house

1. Did you manage dog feces?

🔾 1. Yes 🔾 2. No

1. Do you wash hands before meal?

🔾 1. Yes 🔾 2. No

1. Do you wash hands after touch with soil?

🔾 1. Yes 🔾 2. No

1. Do you wash hands after touch with dog?

🔾 1. Yes 🔾 2. No
